# Contribution of the Unfolded Protein Response (UPR) to the Pathogenesis of Proteasome-Associated Autoinflammatory Syndromes (PRAAS)

**DOI:** 10.3389/fimmu.2019.02756

**Published:** 2019-11-26

**Authors:** Frédéric Ebstein, María Cecilia Poli Harlowe, Maja Studencka-Turski, Elke Krüger

**Affiliations:** ^1^Institut für Medizinische Biochemie und Molekularbiologie, Universitätsmedizin Greifswald, Greifswald, Germany; ^2^Facultad de Medicina Clínica Alemana, Universidad del Desarrollo, Santiago, Chile; ^3^Department of Pediatrics, Baylor College of Medicine, Houston, TX, United States

**Keywords:** proteasome, ER stress, unfolded protein response, mTORC1, TCF11/Nrf1, autoinflammation

## Abstract

Type I interferonopathies cover a phenotypically heterogeneous group of rare genetic diseases including the recently described proteasome-associated autoinflammatory syndromes (PRAAS). By definition, PRAAS are caused by inherited and/or *de novo* loss-of-function mutations in genes encoding proteasome subunits such as *PSMB8, PSMB9, PSMB7, PSMA3*, or proteasome assembly factors including *POMP* and *PSMG2*, respectively. Disruption of any of these subunits results in perturbed intracellular protein homeostasis including accumulation of ubiquitinated proteins which is accompanied by a type I interferon (IFN) signature. The observation that, similarly to pathogens, proteasome dysfunctions are potent type I IFN inducers is quite unexpected and, up to now, the underlying molecular mechanisms of this process remain largely unknown. One promising candidate for triggering type I IFN under sterile conditions is the unfolded protein response (UPR) which is typically initiated in response to an accumulation of unfolded and/or misfolded proteins in the endoplasmic reticulum (ER) (also referred to as ER stress). The recent observation that the UPR is engaged in subjects carrying *POMP* mutations strongly suggests its possible implication in the cause-and-effect relationship between proteasome impairment and interferonopathy onset. The purpose of this present review is therefore to discuss the possible role of the UPR in the pathogenesis of PRAAS. We will particularly focus on pathways initiated by the four ER-membrane proteins ATF6, PERK, IRE1-α, and TCF11/Nrf1 which undergo activation under proteasome inhibition. An overview of the current understanding of the mechanisms and potential cross-talk between the UPR and inflammatory signaling casacades is provided to convey a more integrated picture of the pathophysiology of PRAAS and shed light on potential biomarkers and therapeutic targets.

## Introduction

The ubiquitin-proteasome system (UPS) ensures the degradation of most short-lived intracellular proteins in eukaryotes. Proteins destined for destruction by 26S proteasomes are subjected to a so-called ubiquitination process which relies on a sequence of reactions involving a cascade of ubiquitin thioester complexes catalyzed by E1, E2, and E3 enzymes (1). The 26S proteasome comprises one 20S proteolytic complex and two axially positioned 19S regulatory complexes that exhibit ATPase activity and recognize ubiquitin-protein conjugates (2, 3). In this pathway, regulatory proteins as well as proteins that are misfolded and/or oxidized are typically tagged with K48-linked ubiquitin chains, making them targets for degradation by the 26S proteasome. The 20S proteasome consists of 2 copies each of 7 α and β type subunits, each encoded by a distinct gene. Each β ring possesses three catalytic subunits, i.e.,; β1, β2, and β5 which exhibit caspase-like, trypsin-like, and chymotrypsin-like activities, respectively. Importantly, there are at least two types of proteasomes: (i) the standard form which is present in all cells and (ii) the immunoproteasome which is constitutively present in immune cells such as dendritic cells (DC) or whose expression can be induced in other cell types by interferon (IFN)-α/β or -γ (4–6). Immunoproteasomes distinguish themselves from the standard ones by incorporating the three inducible β-type subunits β1i (LMP2), β2i (MECL1), and β5i (LMP7) which replace the β1, β2, and β5 constitutive subunits, respectively. This picture is further complicated by the recent identification of intermediate-type proteasomes bearing one or two out of the three inducible subunits or tissue-specific subunits such as β5t in the thymus (7–10). In addition, the ability of standard and/or immunoproteasomes to destroy ubiquitin-modified proteins can be greatly influenced by their association with further regulatory complexes including PA28 and PA200 (11–13). It was long believed that the main function of immunoproteasomes was solely restricted to the regulation of MHC class I antigen presentation. However, it recently appeared clear that it was only the tip of the iceberg and that they probably participate in almost all aspects of cell physiology and development (14–16). By controlling the intracellular pool of regulators (e.g., IκB, IRF3), both standard and immunoproteasomes actively participate in the regulation of myriad signaling pathways, including mTOR, the unfolded protein response (UPR) as well as both innate and adaptive immune responses (17–21). By maintaining protein homeostasis in the cell, the UPS also represents a prerequisite for cell integrity, viability and functioning. Recently, an increasing number of loss-of-function (LOF) mutations have been identified in genes encoding proteasome subunits (22–25). Surprisingly, depending on the subunit affected, such alterations result in the development of two seemingly distinct phenotypes, namely: (i) systemic autoinflammation and (ii) neurodevelopmental or neurodegenerative disorders.

## Genomic Alterations Affecting Subunits of the Proteolytic Complex and/or 20S Proteasome Assembly Factors

**Figure 1:**
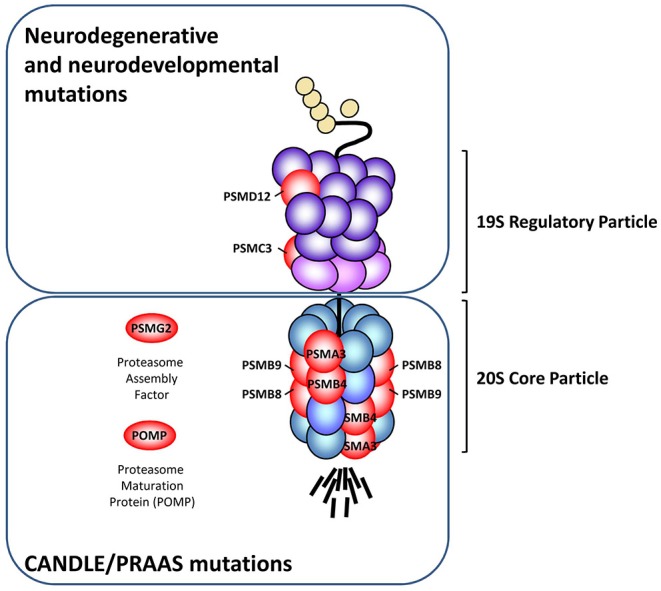
Schematic representation of the proteasome subunits affected by pathogenic loss-of-function mutations. The various proteasome loss-of-function mutations described so far (red) are localized in genes encoding subunits of the 20S core particle (*PSMB8, PSMB9, PSMA3, PSMB4*), 19S regulatory particle (*PSMC3* and *PSMD12*) and the proteasome assembly factors *POMP* and *PSMG2*. While mutations affecting the 20S core particle and/or proteasome assembly factors consistently give rise to an autoinflammatory phenotype known as CANDLE/PRAAS, those affecting the 19S regulatory particle promote neurodegenerative syndromes and/or syndromic intellectual disability, as indicated.

Loss-of-function mutations of the 20S core particle subunits and/or proteasome assembly factors are typically associated with a group of autoinflammatory syndromes referred to as chronic atypical neutrophilic dermatosis with lipodystrophy and elevated temperature (CANDLE) (26–28). The acronym CANDLE was originally brought forward by Torello and al. for the description of autoinflammatory conditions characterized by recurrent fever, skin lesions, lipodystrophy, developmental delay as well as systemic- and neuro-inflammation (29). In the early 2010s, it became clear that CANDLE was caused by pathogenic alterations of the 20S proteasome, as four independent groups identified homozygous missense mutations in the *PSMB8* gene encoding the β5i/LMP7 subunit in patients sharing the same constellation of clinical signs (22–25). In addition to CANDLE and depending on the group that identified the various disease-causing proteasome genes; several different names have been used to describe these disorders. These include joint contractures, muscle atrophy, microcytic anemia, and panniculitis-induced lipodystrophy (JMP), Nakajo-Nishimura syndrome (NKJO), proteasome-associated auto-inflammatory syndrome (PRAAS) and POMP-related auto-inflammation and immune dysregulation disease (PRAID) which all share the same constellation of signs and are all associated with pathogenic mutations in proteasome genes (22–27). In this review, the term CANDLE/PRAAS will be primarily used without distinguishing between the various forms, unless otherwise specified. Importantly, *PSMB8* is not the only disease-causing proteasome gene for CANDLE/PRAAS, as Goldbach-Mansky et al. could identify additional genomic alterations in the *PSMB4, PSMA3, PSMB9* genes encoding the β7, α6 and β1 proteasome subunits, respectively (26) (Figure 1). It also appears that CANDLE/PRAAS is not formally restricted to abnormalities in genes encoding 20S proteasome subunits, since it also includes genetic alterations in proteasome assembly factors (i.e., *POMP* and *PSMG2*) which are proteins involved in the incorporation of these subunits into 20S complexes (27, 28) (Figure 1). Pathogenic variants of proteasome genes causing CANDLE/PRAAS can be either *de novo* or inherited. Monogenic inheritance of CANDLE/PRAAS occurs in an autosomal recessive manner through homozygous or compound heterozygous mutations in the *PSMB8, PSMB4*, and *PSMG2* genes (22–26, 28). A digenic autosomal dominant inheritance pattern due to heterozygous mutations affecting two different proteasome genes (i.e., *PSMA3*/*PSMB8, PSMB4*/*PSMB9*, and *PSMB4*/*PSMB8*) has also been observed in three CANDLE/PRAAS families (26). Up to now, *POMP* is the only form of PRAAS that has been shown to be an autosomal dominant monogenic disease in which the disease-causing variants are *de novo* alterations (27). As expected, one major feature of the pathogenesis of CANDLE/PRAAS shared by all subjects carrying proteasome loss-of-function mutations is the decreased proteasome activity which ultimately results in an aberrant accumulation of cytosolic ubiquitin-protein conjugates (23, 24, 26–28). Intriguingly, the perturbed protein homeostasis detected in these patients is consistently accompanied by manifestations of autoinflammation such as the uncontrolled release of proinflammatory cytokines and the generation and of a typical type I IFN signature with increased transcription rates of IFN-stimulated genes (ISG) including the ubiquitin-like modifier ISG15, the chemokines CXCL9 and CXCL10 (23–28).

## Genomic Alterations Affecting Subunits of the 19S Regulatory Particle

So far, pathogenic mutations in genes encoding subunits of the 19S regulatory particle seem to be less frequent than those affecting the 20S core particle subunits. To the best of our knowledge, only two 19S subunits (i.e., *PSMC3* and *PSMD12*) have been reported in the literature to carry genomic alterations causing diseases (30, 31) (Figure 1). Surprisingly, unlike pathogenic variants of the 20S proteolytic complex, mutants of 19S regulatory particle do not develop CANDLE/PRAAS but mostly promote neurodegenerative or neurodevelopmental disorders. For instance, our work recently revealed that subjects with genomic alterations in the *PSMD12* gene encoding the PSMD12 (i.e., Rpn5) subunit of the 19S regulatory particle do not suffer from CANDLE/PRAAS but syndromic intellectual disability (SID) (31). Like CANDLE/PRAAS subjects, patients with SID carrying *PSMD12* loss-of-function mutations exhibit a decreased turnover of ubiquitin-modified proteins, even though the chymotrypsin-like proteasome activity was not compromised in these individuals. Fascinatingly, the fact that CANDLE/PRAAS subjects also exhibit signs of cognitive impairment supports the notion that both of these syndromes share similarities in their etiology and/or pathogenesis. Nevertheless, whether mutations in 19S proteasome subunits also elicit a type I IFN response remains to be fully determined. The observation that loss-of-function mutations of components of the 19S regulatory particle are not associated with any of the expected CANDLE/PRAAS clinical signs is intriguing but may be partially explained by the fact that, in contrast to the 20S proteasome subunits which are ubiquitously expressed, the 19S proteasome subunits exhibit a more tissue-specific distribution (32). Altogether these data point to a clear association between proteasome dysfunction and type I IFN, even though the mechanisms underlying this cause-and-effect relationship remain obscure.

## Proteasome Dysfunction Is a Danger Signal Alerting the Innate Immune System

The generation of a type I IFN signature in CANDLE/PRAAS subjects carrying proteasome loss-of-function mutations unambiguously associates proteasome impairment with innate immune activation. However, and up to now, the mechanisms by which defective proteasomes promote inflammation in a pathogen-free context remain ill-defined. Sterile activation of the innate immune system usually requires the generation and/or release of endogenous molecules referred to as danger-associated molecular patterns (DAMP) that are sensed by pattern recognition receptors (PRR). Prime examples of DAMP include extracellular purine metabolites such as uric acid and ATP as well as the high-mobility group box (HMGB1) nuclear protein which are released by necrotic and late apoptotic cells following membrane disruption (33). To the best of our knowledge, no DAMP has been specifically associated with proteasome dysfunction so far. Interestingly, the observation that the integrity of the plasma membrane is not necessarily compromised in cells carrying proteasome loss-of-function mutations challenges the classical view that DAMP induce inflammation by acting extracellularly. Rather, it is conceivable that proteasome impairment may result in the intracellular generation of DAMP alerting the immune system. It is indeed highly likely that proteasome dysfunction results in the dysregulation of pathways which are then perceived as danger signals by the innate immune system. Given the central role of proteasomes in many cellular processes, the precise nature of these signals and/or deregulated pathways might be difficult to assess.

## Defective Proteasomes Generate Stress Conditions that Engage the Unfolded Protein Response (UPR)

**Figure 2:**
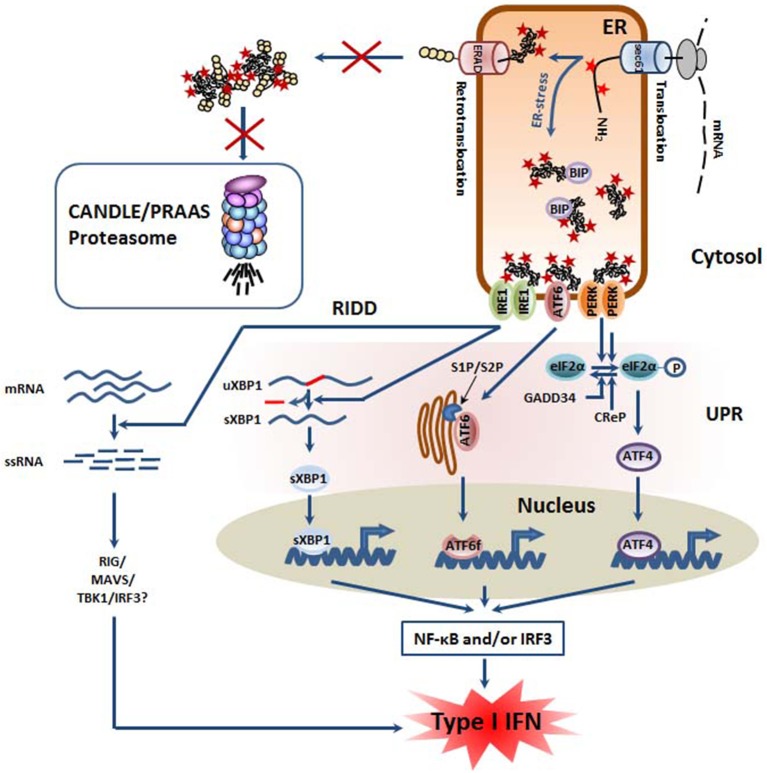
Defective proteasomes in CANDLE/PRAAS subjects provoke ER stress and trigger the so-called unfolded protein response (UPR) which is associated with inflammation. Defective proteasomes impair the ER-associated degradation (ERAD) of misfolded ER proteins, leading to their accumulation within the lumen. Perturbed protein homeostasis is then sensed by the three ER membrane-resident proteins IRE1, ATF6, and PERK which initiate a complex signaling program known as the unfolded protein response (UPR). Thanks to its endonuclease activity, IRE1 promotes the splicing of the untranslated XBP1 mRNA, thus giving rise to spliced XBP1 mRNA species encoding an active transcription factor. The ATF6 protein is transported into the Golgi apparatus where it is subjected to a proteolytic cleavage by the site-1 and−2 proteases (S1P and S2P), thereby generating an ATF6f transcription factor. By promoting the phosphorylation of eIF2α, PERK favors the cap-independent translation of stress proteins such as the ATF4 transcription factor. All transcription factors (sXBP1, ATF6f, and ATF4) activated by the UPR have been shown to initiate sterile inflammation by favoring the activation of the NF-κB and/or IRF3 transcription factors. In addition, IRE1 is implicated in the regulated IRE1-dependent decay (RIDD) pathway in which cellular mRNA are subjected to degradation, thus resulting in the generation of 5′ and 3′ unprotected single stranded RNA which may be sensed as foreign RNA and induce a type I IFN response following their recognition by pathogen recognition receptors (PRR) including RIG-I.

One major consequence of proteasome dysfunction and/or inhibition is the activation of ER-stress pathways as a consequence of impaired associated degradation machinery (ERAD) (34). The ERAD pathway is primarily defined as an ER-localized UPS that ensures the proteasome-mediated degradation of misfolded proteins trafficking in the ER (35, 36). In this process, damaged proteins and/or proteins aberrantly modified are transported from the ER lumen back to the cytosol by a yet unidentified channel (a processed referred to as retro-translocation) prior to subsequent ubiquitination and membrane extraction for degradation by proteasomes. It is understood that ~30% of the total proteins are synthetized at the ER by the secretory pathway and, as such, potential ERAD substrates thereby making ERAD a reliable sensor alarming the cell in case of decreased ability to degrade proteins. By preventing the degradation of ERAD substrates, proteasome inhibition favors the accumulation of aberrant protein species in the ER lumen, thereby triggering a stress response involving multiple pathways known as the UPR (37–39). As illustrated in Figure 2, the UPR itself consists of the activation of ER-resident membrane proteins that are capable of sensing perturbed protein homeostasis in the ER. To date, three of these stress receptors have been identified and include the inositol-requiring enzyme 1 (IRE1), the activating transcription factor 6 (ATF6) and the protein kinase R (PKR)-like endoplasmic reticulum kinase (PERK). It was originally proposed that, due to the constitutive association of their luminal domains with the binding immunoglobulin protein (BiP; also referred to as GRP78/HSP5A), IRE1, PERK, and ATF6 reside as monomers in the ER-membrane in an inactive state under normal conditions. Unbalanced ER protein homeostasis would then result in the physical dissociation of BiP from IRE1, ATF6, and PERK, thereby triggering their activation as well as the subsequent initiation of distinct intracellular signaling cascades known as the three branches or arms of the UPR (40, 41). Recent studies, however, have suggested that the emancipation of BiP from the stress receptors does not necessarily represent an upstream prerequisite for activation of the UPR, as misfolded luminal proteins may also act as direct ligands for IRE1, ATF6, and/or PERK (42–44).

Upon sensing perturbations of protein homeostasis inside the ER, PERK protein undergoes auto-phosphorylation following a monomer-dimer transition which unveils its kinase activity. Phosphorylated PERK can then attenuate global protein synthesis by phosphorylating the eukaryotic initiation factor (eIF2)-α at serine 51, which in turn reduces the intracellular pool of eIF2-guanosine triphosphate-tRNA-methionine ternary complexes (45, 46). This process allows the recovery of protein homeostasis in the ER lumen by preventing further import of nascent proteins into the ER by the polypeptide conducting channel Sec61. Importantly, although initiating a global shutdown of the canonical cap-dependent mRNA translation in the cell, phosphorylated eIF2-α also promotes the selective translation of a small number of transcripts containing a short upstream ORF (uORF) in their 5′ untranslated region (UTR) (47). These mRNAs generally encode proteins for stress adaption and recovery, the most prominent example being the transcription factor 4 (ATF4) whose function mainly resides in the rapid upregulation of genes involved ERAD and/or ER quality control (48–50). Prolonged expression of ATF4 is, however, pathological and represents a no-return point, as it results in the induction of the CCAAT-enhancer-binding protein homologous protein (CHOP), which triggers cell death by suppressing the expression of pro-survival proteins, while favoring that of pro-apoptotic ones (51, 52).

Like PERK, IRE1 becomes active when monomers dimerize und subsequently undergo phosphorylation (53, 54). Unlike PERK, IRE1 exhibits an additional endoribonuclease activity that excises an unconventional 26 base pair intron located in the exon 4 of a transcript encoding a basic leucine zipper (bZIP)-type transcription factor called X-box binding protein (XBP)-1 (55, 56). This mRNA splicing provokes a frameshift that generates a XBP-1 variant which regulates genes involved in protein quality control, among others (57).

Finally, ER stress results in the emancipation of BiP from the type II transmembrane protein ATF6 rendering its Golgi-localization sequences accessible to cytoplasm and thus promoting its subsequent transport to the Golgi-apparatus (58). Following translocation, ATF6 is subjected to a sequential cleavage by the site-1 and−2 proteases (S1P and S2P) releasing a 50 kDa N-terminal fragment (ATF6f) that enters the nucleus and binds to ER stress response elements (59). Genes induced by ATF6f include BiP and XBP-1, thereby supporting the IRE1 arm in a positive feedback loop (60).

The activation of the UPR following proteasome inhibition is particularly prominent in secreting cells and was originally reported in lactacystin-treated pancreatic β-cells (61) before being well-described in response to bortezomib treatment in multiple myeloma (MM) cells (62–64) and other cell types (65, 66). Supporting the notion that the UPR represents a major alarming signal for proteasome dysfunction, Poli et al. have recently shown that CANDLE/PRAAS subjects carrying loss-of-function mutations in the *POMP* gene exhibited higher protein levels of GRP78 than their wild-type counterparts (27). Because GRP78 is induced at the transcriptional level by XBP-1 and ATF6f, these data imply that proteasome dysfunction due to genomic alterations in the *POMP* gene is accompanied by the induction of at least the IRE1 and/or ATF6 branches of the UPR. Whether the UPR participates in mounting the type I IFN response detected in these patients remains, however, to be determined.

## The Unfolded Protein Response (UPR) as a Potential Link Between Proteasome Dysfunction and the Innate Immune System

Interestingly, the UPR seems to play roles beyond simple ER-quality control with important implications for inflammation and metabolism. Over the past few years an increasing body of evidence has suggested a possible relationship between ER-stress and sterile inflammation. Pioneering work of Pahl et al. showed that treatment of 293 cells with well-known ER stress-inducing agents such as tunicamycin (Tm), thapsigargine (Tg), or 2-deoxyglucose causes accumulation of proteins in the ER which was subsequently followed by the nuclear translocation of the transcription factor NF-κB (67). This observation was somehow surprising, since the NF-κB stimuli were initially thought to be extracellular and mainly restricted to threats such as pathogens and/or immune-derived signals including T or B-cell receptor engagement. The fact that NF-κB translocation accompanies ER stress brought forward the concept that unbalanced protein homeostasis may act as a danger signal eliciting inflammation. This assumption was already in line with much of the previous work on Alzheimer and Parkinson diseases showing that abnormal protein aggregation serves a trigger for inflammation and neurodegeneration in the aging brain (68). Moreover, endoplasmic reticulum stress has long been recognized as a key feature in the pathogenesis of monogenic and/or polygenic autoinflammatory syndromes including TNF-associated periodic syndromes (TRAPS) whereby mutations in genes encoding the TNF receptor (TNFR) results in the accumulation of misfolded TNFR species and subsequent activation of the UPR (69–71). Furthermore, autosomal dominant mutations in the vesicle coating protein COP- α, that participates in retrograde transport from Golgi to ER, have also been associated with increased GRP78 and an exacerbated type I interferon response causing COPA syndrome (72, 73) linking the UPR to innate immunity and disease pathogenesis.

Supporting the notion that the UPR is an inducer of sterile inflammation, rescuing ER protein folding by chemical chaperones can significantly reverse inflammation in cells exposed to various ER stress-inducing agents (74–81). Later, the use of knockout models for the ER stress sensors IRE1, ATF6 and PERK or their major downstream targets further confirmed the existence of a cause-and-effect relationship between impaired ER quality control and sterile inflammation. In this regard, Jiang et al. originally reported that PERK deficiency resulted in the inability of mouse embryo fibroblasts to translocate NF-κB into the nucleus when exposed to Tm (82). Interestingly, similar results were obtained by suppressing the IRE1/XBP-1 arm of the UPR by gene silencing, pharmacological inhibition, and/or dominant negative inhibition (83–85). Of note, the functional relationship between IRE1/XBP-1 and NF-κB signaling was recently further underscored by the work of Talty et al. showing that IRE1 itself is capable of promoting inflammasome assembly and subsequent IL-1β processing in response to TLR-4 agonists (86). Likewise, pharmacological inhibition of ATF6 results in decreased NF-κB activity in response to Subtilase cytotoxin (87).

Altogether, these works unambiguously demonstrate that activation of the UPR by either one of its three branches favor the initiation of inflammatory responses by promoting NF-κB nuclear translocation (Figure 2). Interestingly, the molecular mechanisms by which the UPR activates NF-κB do not substantially differ from those initiated by pathogens. The canonical activation pathway of NF-κB following PRR engagement relies on the inactivation by phosphorylation of the inhibitory protein IκBα by the IKK kinase complex. The phosphorylation of IκBα unmasks a lysine residue which is then used for ubiquitin modification and subsequent degradation by the proteasome. The activity of IκBα has been shown to be increased by IRE1 through a process involving the adapting molecule TNF-receptor associated factor (TRAF2) (88–90). TRAF2 recruits ubiquitin E3 ligases such as cellular inhibitor of apoptosis (cIAP) 1 and 2 which subsequently modify the receptor-interacting serine/threonine-protein kinase 1 (RIPK1) with ubiquitin moieties, thereby facilitating the recruitment of the kinases IκB (IKK) and TAK1. Hence, TAK phosphorylates IKK which then undergoes auto-phosphorylation for IKK full activation (91). Because IκBα is less stable than NF-κB, it has been suggested that translational arrest triggered by the PERK-mediated phosphorylation of eIF2α supports NF-κB translocation (88, 92). Other activation pathways of NF-κB by the UPR include the PERK/ATF4 axis and its downstream effector CHOP which has been shown to prevent NF-κB inhibition by the peroxisome proliferator-activated receptor (PPAR)-gamma via sequestration of the CCAAT/enhancer-binding protein (C/EBP)-β transcription factor (93). Conflicting results have been reported regarding the ability of ATF6 to regulate NF-κB, with an earlier study reporting an activating effect of ATF6 on NF-κB (87), while a later one showing an inhibitory function (94).

Interestingly, sterile inflammation mediated by the UPR also includes the activation/phosphorylation of IRF3, a transcription factor inducing type I IFN gene expression which then drives the expression of a wide range of genes referred to as IFN-stimulated genes (ISG) in an autocrine and paracrine manner (95, 96). Like NF-κB, IRF3 is translocated into the nucleus following activation of either one of the three branches of the UPR upon ER stress. The involvement of IRE1 in this process is particularly well-exemplified in XBP-1^−/−^ cells which are less prone to produce type I IFN than their wild-type counterparts following exposure to various ER stress-inducing agents such as Tm (97–100). The contribution of the PERK/ATF4 signaling axis to IRF3 phosphorylation was also evidenced in cells following PERK inhibition and/or gene silencing (101). Similarly, blocking ATF6 activation by inhibiting S1P results in decreased IRF3 phosphorylation in response to Tg and oxygen-glucose deprivation (102), suggesting that the ATF6 arm of the UPR has also the potential to drive a type I IFN response even in a pathogen-free cellular context.

Interestingly, the IFN-β promotor is endowed with four transcription factors binding sites (referred to as positive regulatory domains), one of which allowing the binding to the NF-κB transcription factor (103). In this regard, previous studies have reported an essential role for NF-κB in the induction of type I IFN (104). The observation that CANDLE/PRAAS is frequently associated with sustained expression of typical NF-κB-responsive genes such as IL-6 points to a persistent activation of this transcription factor in these patients. Whether either one or both of NF-κB and IRF3 are involved in the generation of the type I IFN signature associated with CANDLE/PRAAS is currently unknown and further investigations addressing this point should help deciphering the nature of the UPR engaged in these patients.

Of particular interest in that regard is also IRE1 from the UPR whose phosphorylation following ER-stress activates its ribonuclease activity to remove an intron of the XBP-1 mRNA, thereby producing a potent transcription factor involved in the upregulation of genes encoding ER chaperones and/or ERAD components. In addition to activating XBP-1, IRE1 has been linked to the degradation of various cytosolic mRNA that accumulates due to the protein biosynthesis stop mediated by eIF2-α phosphorylation, a process known as regulated IRE1-dependent decay (RIDD) (105, 106). Most importantly, by processing RNA, RIDD also generates short single stranded (ss) RNA devoid of protection sequences at 5′ and 3′ ends which are then rapidly degraded by cellular exoribonucleases (107). Alternatively and depending on their amounts, these short ssRNA may be sensed by the retinoic acid inducible gene (RIG)-1 cytosolic RNA, thereby initiating a signaling cascade involving the mitochondrial antiviral-signaling protein (MAVS) and IRF3 which eventually leads to the production of type I IFN (108). Supporting this notion, Studencka-Turski and colleagues show in this issue of *Frontiers in Immunology* that proteasome inhibition in microglia cells results in increased expression of IFN-responsive genes in an IRE1-dependent manner (submitted manuscript). Nonetheless, it has been recently shown that RIDD also mediates the degradation of the microRNA (miR)-146a and−155 which in turn result in exacerbated LPS signaling in dermal fibroblasts in subjects suffering from TRAPS (109). Based on this finding, one can therefore not exclude that the ability of RIDD to promote inflammation in PRAAS may also occur through RIG-1-independent mechanisms.

In any case, these data provide a conceptual framework for explaining the onset of autoinflammation in subjects suffering from proteasome loss-of-function mutations, even though this point remains to be fully demonstrated in patients' cells.

## PERK Is Part of the Integrated Stress Response (ISR) Which Represses Lipid Biogenesis

The observation that CANDLE/PRAAS patients exhibit severe signs of both lipodystrophy and panniculitis supports a cause-and-effect relationship between proteasome loss-of-function mutations and altered lipid metabolism (26). Major regulators of lipid biogenesis include the mTORC1 signaling pathway whose dysfunction has been frequently associated with the pathogenesis of various inflammatory diseases (110). By inhibiting the phosphatidic acid phosphatase Lipin-1, mTORC1 supports the upregulation of genes involved in cholesterol biosynthesis by controlling the activity and trafficking of sterol regulatory element-binding proteins (SREBP) (111). Activation of mTORC1 is typically achieved by anabolic stimuli including growth factors, oxygen, energy, and/or free amino acids (112).

Remarkably, mTORC1 is capable of sensing amino acid deficiency following activation of the general control non-derepressible 2 (GCN2) kinase during the so-called integrated stress response (ISR) (113, 114). Under starvation, GCN2 undergoes auto-phosphorylation and subsequent activation upon binding to uncharged tRNA. Like PERK, GCN2 mediates the phosphorylation of the eukaryotic translation initiation factor 2α (eIF2α), thereby promoting the cap-independent translation of various stress proteins including ATF4. In turn, ATF4 decreases mTORC1 activity by inducing the transcription of several mTORC1 inhibitors such as the eukaryotic translation initiation factor 4E-binding protein 1 (4E-BP1) (115) as well as the Sestrin 2 (SESN2) (116), tribbles homolog 3 (TRIB3) (117), and regulated in development and DNA damage response 1 (REDD1) (118) proteins.

**Figure 3:**
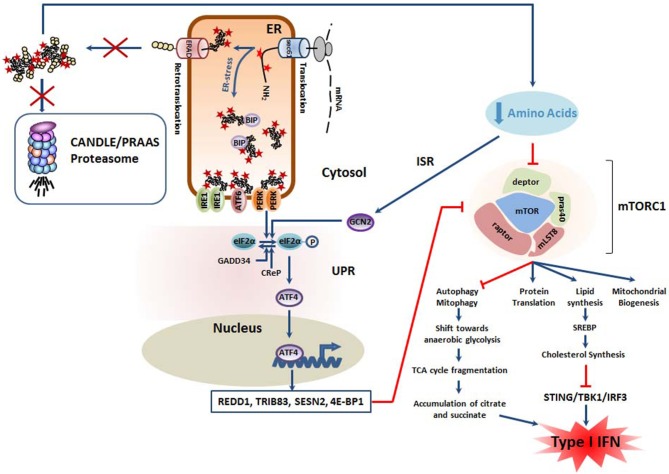
Mutant proteasomes from CANDLE/PRAAS subjects have a potential pro-inflammatory potential due to the negative impact they exert on mTORC1. The decreased degradation capacity of cells carrying CANDLE/PRAAS proteasomes results in amino acid deficiency which causes engagement of the integrative stress response (ISR). In this pathway, the GCN2 kinase intersects with the UPR by phosphorylating eIF2α, thus promoting the upregulation of ATF4 and the subsequent transcription of various inhibitors of the mTORC1 complex including REDD1, TRIB83, SESN2 and 4E-BP1, as indicated. In addition, the drop in intracellular levels of free amino acids is directly sensed by mTORC1, resulting in its downregulation. Decreased mTORC1 activity is accompanied by excessive mitophagy and a potential metabolic shift from oxidative phosphorylations to anaerobic glycolysis. This results in the fragmentation of the tricarboxylic (TCA) cycle and parallel accumulation of pro-inflammatory metabolites as citrate and succinate. Besides, the suppression of mTORC1 signaling leads to cholesterol deficiency which has been shown to act as a danger signal initiating a type I IFN response following sensing by STING1.

Given that proteasome-mediated protein degradation represents a major source of peptides which can be further degraded into amino acids by various peptidases (119), it is highly likely that CANDLE/PRAAS subjects carrying proteasome loss-of-function mutations suffer from a reduced intracellular pool of free amino acids and engage, in addition to the UPR, the ISR. In this situation, one would therefore expect a decreased activation of mTORC1 and a subsequent drop of all anabolic processes including cholesterol biosynthesis (Figure 3). This point is of great importance, since recent evidence suggests that cholesterol deficiency elicits a type I IFN response. It has been indeed recently reported that a reduced cellular cholesterol flux may trigger a type I IFN response through a STING-dependent mechanism (120). It is therefore conceivable, that CANDLE/PRAAS patients may spontaneously engage type I IFN signaling because of perturbed cholesterol homeostasis occurring as a consequence of decreased mTORC1 activity following activation of the UPR and/or the ISR (Figure 3).

Strikingly, the UPR itself also affects cholesterol synthesis by two additional mTORC1-independent mechanisms. Indeed, prominent RIDD substrates include transcripts encoding enzymes involved in cholesterol biosynthesis such as the phosphomevalonate kinase (PMVK) and squalene monooxygenase (SQLE), which undergo rapid degradation following IRE1 activation (121). Furthermore, GRP78 which is upregulated by all three arms of the UPR has been reported to reduce the expression of SREBP2, a transcription factor required for the induction of genes of cholesterol biosynthesis (122). Altogether, these studies point to strong cause-and-effect relationship between the UPR and impaired cholesterol synthesis. Whether cholesterol deficiency is a trigger of the type I IFN response in CANDLE/PRAAS patients remains, however, to be determined.

## Major Targets of the ISR Include Mitochondria

In addition to promoting anabolic processes, mTORC1 is a critical inhibitor of autophagy and thus the PINK1/PARK2 axis which targets damaged mitochondria for cargo-specific autophagy (123). This process also referred to as “mitophagy” is essential for preserving mitochondria homeostasis and preventing the harmful leakage of mitochondrial DNA (mtDNA) and/or radical oxygen species (ROS) into the cytosol. On the other hand, persistent mitophagy may result in a net loss of mitochondria and trigger a metabolic shift toward anaerobic glycolysis. As illustrated in Figure 3, this would be accompanied by the accumulation of tricarboxylic acid cycle (TCA) intermediates (i.e., citrate, succinate) which favor the acquisition of a pro-inflammatory phenotype (also referred to as M1 phenotype in macrophages) by various mechanisms (124). Based on the assumption that mTORC1 activity is decreased following proteasome dysfunction, it is tempting to speculate that excessive mitophagy contribute to autoinflammation in CANDLE/PRAAS subjects.

Alternatively, and independent of mTORC1, it is also conceivable that mitochondria of CANDLE/PRAAS subjects face a Ca^2+^ challenge because of sustained ER stress. Calcium, which is a critical regulator of mitochondria function, is under normal conditions, stored in the ER lumen and transiently released into the cytosol in response to specific stimuli. Calcium export from the ER into the cytosol mostly occurs through the 4,5-triphosphate (IP3) receptor (IP3R) excitable channel, while calcium import from the cytosol into the ER is controlled by sarcoendoplasmic reticulum Ca^2+^-transport ATPases (SERCA) (125). Interestingly, it is well-established that the Ca^2+^ flux between the ER and the cytosol is perturbed under ER stress conditions (126). For instance, it has been shown that the PERK/ATF4/CHOP axis of the UPR induces the transcription of a SERCA1 splice variant (S1T) which is unable to pump Ca^2+^ into the ER, resulting in an accumulation of cytosolic Ca^2+^ (127). In addition, the very same arm of the UPR mediates the upregulation of the ER oxidoreductin (ERO)-1α, which in turn activates IP3R, thereby aggravating cytosolic Ca^2+^ overload (128–130). The increased Ca^2+^ concentration may then activate the Ca^2+^/calmodulin-dependent protein kinase II (CaMKII) which intersects with the c-Jun N-terminal kinase (JNK) pathway resulting in the generation of reactive oxygen species (ROS) via the induction of the NADPH oxidases (NOX)-1 and−2 (126, 131). Strikingly, the calcium-CaMKII-NOX2 pathway has been shown to activate the protein kinase R (PKR) (132), thereby unveiling a potential additional link between ER stress and type I IFN production. Indeed, PKR is a serine/threonine kinase which normally undergoes activation by autophosphorylation upon sensing of double stranded RNA and whose functions include, beside the phosphorylation of eIF2α, the initiation of a poorly understood signaling cascade leading to type I IFN responses (133–136). Alternatively, the increased cytosolic Ca^2+^ may affect mitochondrial function and/or permeability and result in the release of ROS and mtDNA, which in turn activate NF-κB and IRF3, respectively (137).

## TCF11/Nrf1: the (Still) Unrelated ER Partner of the UPR

**Figure 4:**
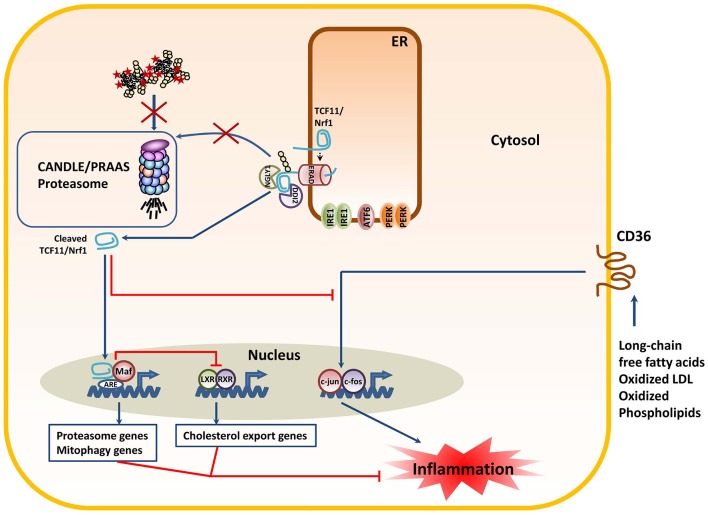
The decreased proteasome activity in subjects with CANDLE/PRAAS triggers the processing of the TCF11/Nrf1 transcription factor with anti-inflammatory consequences. Proteasome dysfunction due to loss-of-function mutations prevents the rapid degradation of the TCF11/Nrf1 protein by ERAD, thus resulting in its de-glycosylation by NGLY1 and subsequent proteolytic cleavage by DDI2. The C-terminal TCF11/Nrf1 processed fragment enters the nucleus to form heterodimers with small Maf proteins and induce the transcription of proteasome and mitophagy genes. In addition, processed TCF11/Nrf1 promotes the trans-respression of LXR-responsive genes such as those involves in cholesterol export. Both of these events are considered anti-inflammatory, as they prevent the harmful leakage of mitochondrial DNA into the cytosol and preserve cholesterol homeostasis in the cell. The anti-inflammatory properties of TCF11/Nrf1 are further exemplified by its capacity to suppress the signaling cascade triggered by the CD36 upon binding to pro-inflammatory lipid molecules.

Besides the UPR, another key compensatory mechanism originating from the ER for proteasome dysfunction is the processing of the cap-n-collar (CNC) basic leucine zipper (bZIP) protein TCF11/Nrf1. TCF11/Nrf1 (also known as the nuclear factor erythroid-derived 2-related factor 1 encoded by the *NF2EL1* gene) is a transcription factor residing in the ER-membrane which regulates gene expression through the antioxidant/electrophile response element (ARE) in a broad range of gene promoters in response to various stimuli (138). Unlike the UPR mediators IRE1, ATF6 and PERK, TCF11/Nrf1 does not constitutively bind to BiP and does not respond to perturbed ER protein-folding homeostasis *per se*. Rather, TCF11/Nrf1 seems to perceive signals emerging from the cytosol, mitochondria, or the ER membrane itself. Typical inducers of TCF11/Nrf1 include proteasome inhibitors (139–141), ROS (142), and/or cholesterol excess (143). In unstressed cells, TCF11/Nrf1 is a short-lived protein that is constitutively subjected to proteasome-mediated degradation following retro-translocation into the cytosol via ERAD. As shown in Figure 4, the events leading to TCF11/Nrf1 activation are complex and involve a de-glycosylation step by the N-glycanase NGLY1 (144) prior to a proteolytic cleavage by the aspartyl protease DDI2 (145) and subsequent liberation of a C-terminal processed fragment that enters into the nucleus. After translocation, TCF11/Nrf1 forms heterodimers with small Maf proteins to bind promotors of an array of genes (146). Target genes of TCF11/Nrf1 include all 19S and 20S proteasome subunits with exception of those of the immunoproteasomes (i.e., β1i, β2i, and β5i) (139–141). Hence, it is understood that TCF11/Nrf1 activation following proteasome inhibition is a critical process aiming to restore protein homeostasis by inducing the synthesis of new proteasomes. Interestingly, TCF11/Nrf1 also induces the transcription of genes involved in mitophagy (147), thereby preventing the access of inflammatory mtDNA and/or ROS to the cytosol. Also, thanks to its ability to suppress the CD36 signaling pathway, the processing of TCF11/Nrf1 has been shown to attenuate lipid-mediated inflammation (143). For these reasons, unlike the UPR which activates proinflammatory pathways, TCF11/Nrf1 seems to protect the cells against inflammation. Interestingly and as expected, CANDLE/PRAAS patients are enriched with processed TCF11/Nrf1 (27, 142), indicating that both pro- and anti-inflammatory responses are engaged in cells with proteasome loss-of-function mutations. The fact that CANDLE/PRAAS subjects develop autoinflammation strongly suggests an imbalance of the UPR and TCF11/Nrf1 responses in detriment of TCF11/Nrf1. One interesting and decisive candidate initiating disequilibrium between the UPR and TCF11/Nrf1 might be mTORC1 whose activity has been shown to upregulate TCF11/Nrf1 gene expression (148). Given that mTORC1 activity requires proteasome activity (149), it is seductively easy to imagine that TCF11/Nrf1 is quantitatively much weaker induced than its IRE1, ATF6, and PERK ER counterparts in CANDLE/PRAAS patients.

## Concluding Remarks

A prerequisite for understanding the pathogenesis of CANDLE/PRAAS is the identification of the signaling pathways initiated by proteasome disruption. Although the cellular and molecular events triggered by proteasome loss-of-function mutations are likely to be complex and diverse, a growing body of evidence clearly identifies the UPR as one of these pathways. The capacity of the UPR of promoting the activation of the transcription factors NF-κB and/or IRF3 may explain the inflammatory phenotype of CANDLE/PRAAS patients. Nevertheless, the intersection of the UPR with the ISR as well as the convergence of both of these responses to mTORC1 raises the possibility of the implication of a general metabolic dysregulation in the acquisition of a type I IFN signature. Since mTORC1 activity is damped by both the UPR and ISR, such deregulation could imply an increased degradation of mitochondria by autophagy and a subsequent shift from oxidative phosphorylation to glycolysis with accumulation of pro-inflammatory TCA metabolites. Alternatively, it is also conceivable that the decreased activity of mTORC1 leads to a cholesterol deficiency that is sensed as a danger signal by innate immune receptors. In any case, the role of metabolism in this pathogenesis of CANDLE/PRAAS warrants further investigations.

Most importantly, proteasome dysfunction also engages a TCF11/Nrf1-based signaling program, which in contrast to the UPR, seems to possess anti-inflammatory potential. It is likely that an imbalance between TCF11/Nrf1 and the UPR might reflect a key aspect of CANDLE/PRAAS pathogenesis. The further identification of factors regulating this fragile equilibrium might help deciphering the mechanisms underlying the pathophysiology of CANDLE/PRAAS or other proteasome-related disorders.

## Author Contributions

FE conceived, wrote, and edited the manuscript. MP, MS-T, and EK participated in data analysis and provided intellectual input into the manuscript. Figures were designed by FE.

### Conflict of Interest

The authors declare that the research was conducted in the absence of any commercial or financial relationships that could be construed as a potential conflict of interest.
